# Application of machine learning in combination with mechanistic modeling to predict plasma exposure of small molecules

**DOI:** 10.3389/fsysb.2023.1180948

**Published:** 2023-06-20

**Authors:** Panteleimon D. Mavroudis, Donato Teutonico, Alexandra Abos, Nikhil Pillai

**Affiliations:** ^1^ Quantitative Pharmacology Research, DMPK, Sanofi, Cambridge, MA, United States; ^2^ Translational Medicine and Early Development, Sanofi, Chilly-Mazarin, France; ^3^ Commercial Data Science, Sanofi, Barcelona, Spain

**Keywords:** machine learning, drug discovery, pharmacokinetics, PBPK, model-based drug development, artificial intelligence, QSAR

## Abstract

Prediction of a new molecule’s exposure in plasma is a critical first step toward understanding its efficacy/toxicity profile and concluding whether it is a possible first-in-class, best-in-class candidate. For this prediction, traditional pharmacometrics use a variety of scaling methods that are heavily based on pre-clinical pharmacokinetic (PK) data. We here propose a novel framework based on which preclinical exposure prediction is performed by applying machine learning (ML) in tandem with mechanism-based modeling. In our proposed method, a relationship is initially established between molecular structure and physicochemical (PC)/PK properties using ML, and then the ML-driven PC/PK parameters are used as input to mechanistic models that ultimately predict the plasma exposure of new candidates. To understand the feasibility of our proposed framework, we evaluated a number of mechanistic models (1-compartment, physiologically based pharmacokinetic (PBPK)), PBPK distribution models (Berezhkovskiy, PK-Sim standard, Poulin and Theil, Rodgers and Rowland, and Schmidt), and PBPK parameterizations (using *in vivo*, or *in vitro* clearance). For most of the scenarios tested, our results demonstrate that PK profiles can be adequately predicted based on the proposed framework. Our analysis further indicates some limitations when liver microsomal intrinsic clearance (CLint) is used as the only clearance pathway and underscores the necessity of investigating the variability emanating from the different distribution models when providing PK predictions. The suggested approach aims at earlier exposure prediction in the drug development process so that critical decisions on molecule screening, chemistry design, or dose selection can be made as early as possible.

## Introduction

The prediction of the pharmacokinetic (PK) exposure of drugs is critical to understanding their behavior, with researchers striving for optimal concentration *versus* time profile at the site of action to reach both efficacy and a suitable safety profile at a given dose and regimen (Hutchinson and Kirk, 2011; Khanna, 2012; Scannell et al., 2012; Schuck et al., 2015; Waring et al., 2015; Pammolli et al., 2020; Davies et al., 2020). PK profile is a function of both a drug’s intrinsic molecular properties such as lipophilicity, solubility, and chemical reactivity and an organism’s physiological characteristics that ultimately drive its absorption, distribution, metabolism, and excretion (ADME) (Lucas et al., 2019). Although there have been major efforts to using machine learning (ML) to predict the intrinsic properties of compounds for *de novo* molecular design, ML-based predictions of *in vivo* PK dynamics has been much less prominent (Obrezanova et al., 2022). A possible reason for this is that generating a large quantity and high quality of PK exposure data is far more costly and difficult than *in vitro* assays that can be run more easily to characterize a compound’s intrinsic properties (Hughes et al., 2011; Bender and Cortes-Ciriano, 2021). This hinders the application of ML in predicting PK dynamics.

Current approaches for predicting *in vivo* PK mainly involve mathematical models that build upon the classical foundations of pharmacology (Jusko, 2013; Ayyar and Jusko, 2020). These models can be categorized as non-compartmental, compartmental, and physiological. Noncompartmental analysis (NCA) is a useful analysis of concentration vs. time data to assess a drug’s maximum concentration (C_max_), area under the curve (AUC), clearance (CL), and steady-state volume of distribution (Vdss) for the preliminary assessment of properties such as linearity and stationarity. Compartmental models are improving the insights into distribution properties of drugs by incorporating “black box” compartments to capture the different slopes of the PK profile. Physiologically based PK (PBPK) models retain a model structure that is based on the physiology of the species of interest, with parameters divided into those representing a compound’s intrinsic properties and those assigned to the physiological measurements of the body (e.g., blood flow and organ size) (Rowland et al., 2011). Based on their structure, the use of ML approaches is increasingly appealing for predicting the input parameters for PBPK models to not only accelerate the development of robust PBPK-based predictions but also to save substantial resources and become an alternative approach to traditional *in vivo* data-based modeling (Hosea and Jones, 2013).

Several recent studies have used ML approaches to predict *in vivo* PK parameters such as C_max_, AUC, and Vdss (Schneckener et al., 2019; Wang et al., 2019; Ye et al., 2019; Feinberg et al., 2020; Kosugi and Hosea, 2020; Kosugi and Hosea, 2021; Lombardo et al., 2021; Miljkovic et al., 2021), while others studies have incorporated ML and PK models to predict PK profiles (Hosea and Jones, 2013; Schneckener et al., 2019; Antontsev et al., 2021; Chen et al., 2021; Chou and Lin, 2022). Predicting a drug’s PK profile rather than PK parameters can be advantageous, especially regarding its relationship to efficacy and toxicity. This relationship is not easy to correlate to a specific PK parameter but rather to its concentration time course at the site of action for the time the drug interacts with its target. In addition, compared to predicting PK solely through data-based methods, systems-based models such as PBPK can facilitate both the prediction of PK profile for different species and for a different tissue from that used to train the PBPK model. Both extrapolations can take advantage of the physiological basis of the model. However, the information needed to inform a systems-based model like PBPK is not always available in the early stages of drug development, and these models often incorporate numerous assumptions that they cannot be appropriately addressed.

We here evaluate the framework for using ML in combination with mechanism-based modeling (PK and PBPK) to predict the PK profile of intra-venous (IV) administration of small molecules in rats for 1 mg/kg dose. We thus evaluate several test cases where different types of mathematical models (1-compartment PK model, PBPK), different inputs to the models, and different distribution assumptions are considered. The overall goal of this proof-of-concept work is to evaluate whether using ML-derived parameters in tandem with PK/PBPK modeling can result in reasonable exposure predictions and to demonstrate the feasibility of conducting these predictions early after discovery, only knowing the structure of the molecule. ML algorithms developed here are meant to be continuously optimized by the incorporation of new datasets.

## Materials and methods

### Modeling framework and input parameters

**FIGURE 1:**
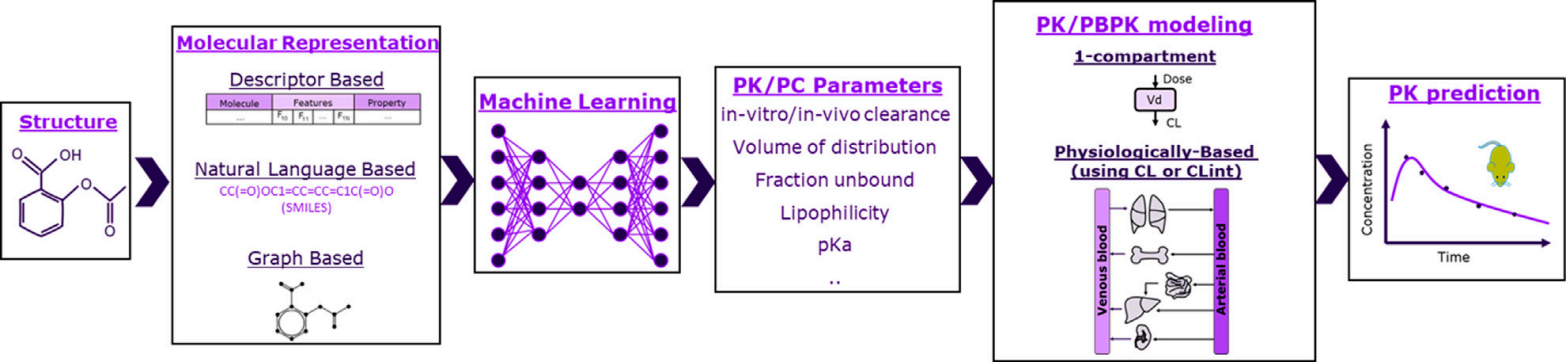
Schematic of the framework studied in this work. Molecules were represented as molecular fingerprints (bit vector), descriptors, or graphs. Machine learning was then used to predict pharmacokinetic (PK) and physicochemical (PC) parameters based on molecular representations. PK/PC parameters were finally inserted to either 1-compartment or physiologically based models to predict rats’ exposure.

The schema of the framework used in this work is shown in Figure 1, where ML is used to predict PK and physicochemical (PC) parameters that are then used as input to mechanistic models to ultimately predict rat plasma exposure for IV administration. We therefore investigated 1-compartment and PBPK models. For PBPK modeling, we explored two parameterizations using as input either *in vivo* or microsomal intrinsic clearance (CL or CL_int_), and we tested five distribution models: Berezhkovskiy (Berezhkovskiy, 2004), PK-Sim standard (Willmann et al., 2005), Poulin and Theil (Poulin and Theil, 2000), Rodgers and Rowland (Rodgers et al., 2005), and Schmidt (Schmitt, 2008). The parameter inputs required for the different models are shown in Table 1.

**TABLE 1:** Mechanistic models developed and the respective parameters required for IV administration PK prediction. For the physiologically based model, we used either *in vivo* clearance (CL) measured with NCA or intrinsic clearance (CL_int_) from liver microsomes.

Models developed	Input required
1-compartment model	*In vivo* clearance (CL)
	Volume of distribution (Vdss)
Physiologically based model	Clearance (CL, or CLint)
	Number of halogens
	Lipophilicity (logP)
	pKa (mostly acidic)
	pKa (mostly basic)
	Fraction unbound (Fu)
	Molecular weight (MW)

### Data used for ML and PK/PBPK model development

To ensure data consistency and to mitigate variability due to assay choice, historical data generated exclusively in the Sanofi Boston site were used. These data were derived from two datasets: the compound property dataset, where structural, PC, and PK parameters are stored, and the PK profile dataset, where concentration time profiles for the different compounds are saved. The compound property dataset consisted of 530 compounds for which *in vivo* rat CL and Vdss were calculated based on NCA of 1 mg/kg IV administration in rats, and 451 compounds for which CL_int_ was calculated based on *in vitro* microsomal experiments. There were 459 and 324 compounds with available information for pka (most acidic) and pka (most basic), respectively, and only 188 compounds for which Fu data were available. SMILES (simplified molecular-input line-entry system) string format was available for each compound. The PK profile dataset consisted of 397 compounds for which we had rat PK profile (concentration vs. time data) for 1 mg/kg IV administration in rats. A total of 637 unique compounds were obtained after combining the aforementioned datasets, of which 61 compounds were found to be common across all. As shown by chemical similarity distribution based on Tanimoto score (Supplementary Figure S1), most compounds in our dataset differed from each other.

### Machine learning methodology

As a first step in our proposed framework, ML models were built to predict the parameters required as input to mechanistic models. Details about the data used to develop these models are provided in Table 2. In-house ML models were built for fraction unbound (Fu), *in vivo* CL, *in vivo* Vdss, CL_int_, pKa (mostly acidic), and pKa (mostly basic). For molecular weight and lipophilicity (logP), they Python RDkit package was utilized to generate predictions (Swain, 2016b; Cheminformatics, 2022; RDKit, 2022; RDKit, 2023).

**TABLE 2:** Metrics for different models utilized to generate final prediction. Ten-fold cross-validation was performed on the training set for hyperparameter optimization (see Supplementary Table S2 for MAPE scores of cross-validation datasets). Performance metrics are reported for the best model.

Parameter	Size of test data	Size of train data	Algorithm	MAPE	RMSE	Unit
CL	61	469	XGboost on fingerprints	0.82	15.74	ml/min/kg
Vdss	61	469	XGboost on descriptors	0.57	961	ml/kg
CL_int_	61	390	XGboost on descriptors	0.75	30.6	ul/min/mg
pKa (most basic)	61	398	Support vector regression on fingerprints	0.28	1.47	
pKa (most acidic)	61	263	Random forest on fingerprints	0.054	1.2	
Fu	61	127	Random forest on descriptors	1.11	0.052	
logP	61		rdkit	0.4767	1.215,068	
MW	61		rdkit	1.84E-05	0.008761	g/mol

SMILES strings—one of the most widely used ways to represent a molecule—were used as input to the ML model. For most structures, one part of the SMILES string consists of salt, and another part represents the base chemical structure for the compound; thus, initial data cleaning was performed prior to training the ML models. In the data cleaning process, salts were stripped from the molecules. In the pre-processing step, the SMILES strings were converted into their canonical forms, and SMILES strings were standardized using the MolVS package (Swain, 2016a) in Python. The SMILES strings, prior to feeding into the algorithm, were transformed into molecular descriptors (200 structure-based descriptors (RDKit, 2022)), molecular fingerprints (Morgan fingerprints, 1024 bit), and graphs (undirected graph represented as a 3-tuple (atom_features, bond_features, and pair_indices)) (Kensert, 2021) to test which molecular representation may help generate models (by testing different algorithms) with better accuracy (lower root-mean squared error (RMSE) or mean absolute percent error (MAPE)). ML models were developed by splitting the dataset into test and train sets. Validation was performed using ten-fold cross-validation on the training set. During cross-validation, hyperparameter optimization was also performed : for random forest algorithm, they were the number of estimators and max depth; for XGBoost, they were the number of estimators, subsample, colsample by tree, and learning rate; for support vector regression, they were kernel, gamma, regularization parameter, and epsilon (Pedregosa et al., 2011); for message passing neural network, they were the number of layers, number of neurons, and activation function (Kensert, 2021). Different molecular representations (molecular fingerprints, descriptors, and graphs (specifically for message-passing neural networks (MPNN))) were initially tested to determine which molecular representation had the best performance metrics (RMSE, MAPE) for our datasets. Different ML algorithms (random forest (Liaw and Wiener, 2002), support vector regression (SVR) (Awad and Khanna, 2015), XGboost (Chen and Guestrin, 2016), and MPNN (Gilmer et al., 2020)) were also tested to see which had the best performance metrics (Supplementary Table S1). To avoid biased predictions (predictions of PK profile generated for compound available in training set) and have uniform predictions generated from one compartment model and PBPK model, 61 compounds for which we had all associated data (PK, PC parameters, and PK profile) were used in the test set for all the models. Based on data availability, the rest of the compounds were used in the training set (see Table 2). Different algorithms, including both classical (random forest, XGboost, and SVR) and deep learning (MPNN) algorithms were tested on different combinations of molecular representation (fingerprints, descriptors, and graphs) to identify which combination of algorithm and molecular representation worked best for the data available (Table 2). Root-mean square error and mean absolute percent error were used to determine the final model.

### PK/PBPK model characteristics and assumptions

#### 1-Compartment modeling

The 1-compartment model assumes that the distribution of the drug takes place homogeneously in a single volume of distribution (Vdss), and its clearance is mediated by a unique rate of elimination (CL) following first-order kinetics (Talevi and Bellera, 2021). The one-compartment model was developed using MATLAB R2019a.

#### Physiologically based pharmacokinetic modeling

A whole-body physiologically based pharmacokinetic (PBPK) model was used to simulate the plasma concentration profiles for the same 61 drug compounds which were available in the test set of ML models. The PBPK model structure and its assumptions are detailed elsewhere (Willmann et al., 2003). Briefly, this model incorporates multiple compartments that represent physiologically relevant body tissues. The parameters of the model are related to the PC drug properties (e.g., lipophilicity and MW) and to physiological information (e.g., tissue volumes and blood flows) that are combined to predict the time course of the drug in the most relevant organs of drug distribution, metabolism, and excretion. Drug properties are used to predict tissue permeabilities and partition coefficients which, in turn, are used to predict the drug distribution in the different tissues. Since several distribution models are available to predict the drug partition coefficients, all the distribution models available were used to generate the drug concentrations, and respective variability was evaluated. PK/PC parameters were included in the simulation as measured or predicted from ML algorithms, depending on the simulation scenario. Simulations with both *in vitro* and *in vivo* clearance parameters were used to compare the predictability of these two parameters. *In vitro* clearance values were used as direct input into the PBPK model after conversion to specific clearance (intrinsic clearance normalized to the liver volume), while plasma clearances were converted into intrinsic clearances using the well-stirred model equation. This conversion was performed using PBPK modeling software. The PBPK model was implemented in PK-Sim, part of the Open Systems Pharmacology Suite version 11.0 (https://www.open-systems-pharmacology.org/). R version 4.2.0 (R Foundation for Statistical Computing) was used to perform the simulations.

## Results

**FIGURE 2:**
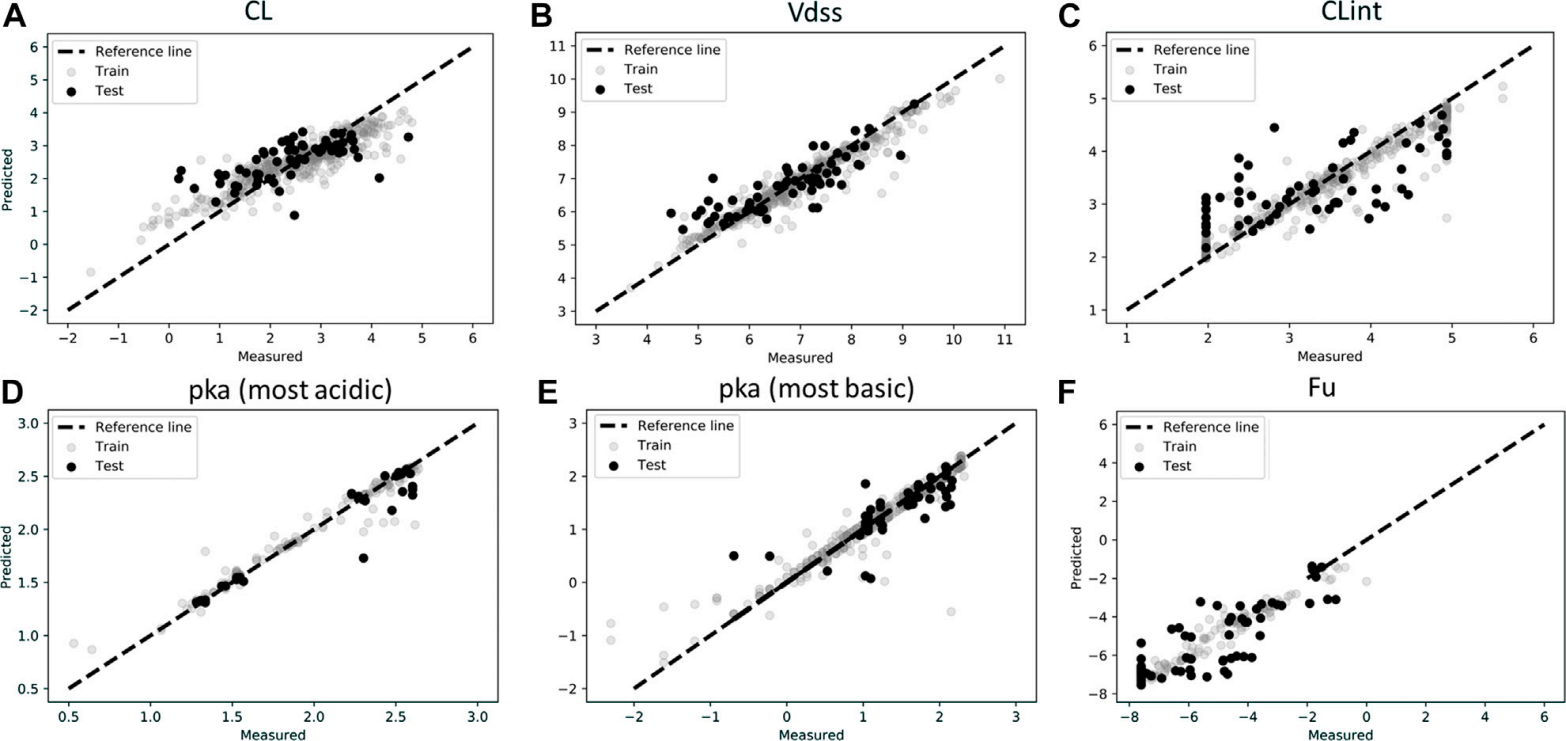
Scatter plots of observations (experimentally measured data) and predictions for test and train data in log scale for **(A)** Clearance (CL), **(B)** Volume of distribution (Vdss), **(C)** Intrinsic Clearance (CLint), **(D)** pka (most acidic), **(E)** pka (most basic), **(F)** Fraction unbound (Fu). Model predictions were based on best model as described in Table 2.

Figures 2A–F show the measured vs. predicted values for each of the parameters tested. ML predictions for CL, Vdss, CLint, pKa (most acidic), and pKa (most basic) were found to be reasonable, with less than two-fold error (MAPE <1) for the final model. MAPE corresponding to fraction unbound was 1.11, which may be due to the low amount of data in the training set (Figure 2F). It was found for this dataset that deep learning methods were less accurate than the classical approach, perhaps due to a low amount of data used in the training set. Finally, it was observed that the MPNN model (deep learning) converged to the mean value of the parameter and hence was not able to capture the distribution spread. Performance metrics of the final model used for ML predictions are provided in Table 2, and performance metrics of different models tested for this analysis are provided in Supplementary Table S1. A combination of MAPE and RMSE were utilized to select the best model for each parameter (Supplementary Table S1).

**FIGURE 3:**
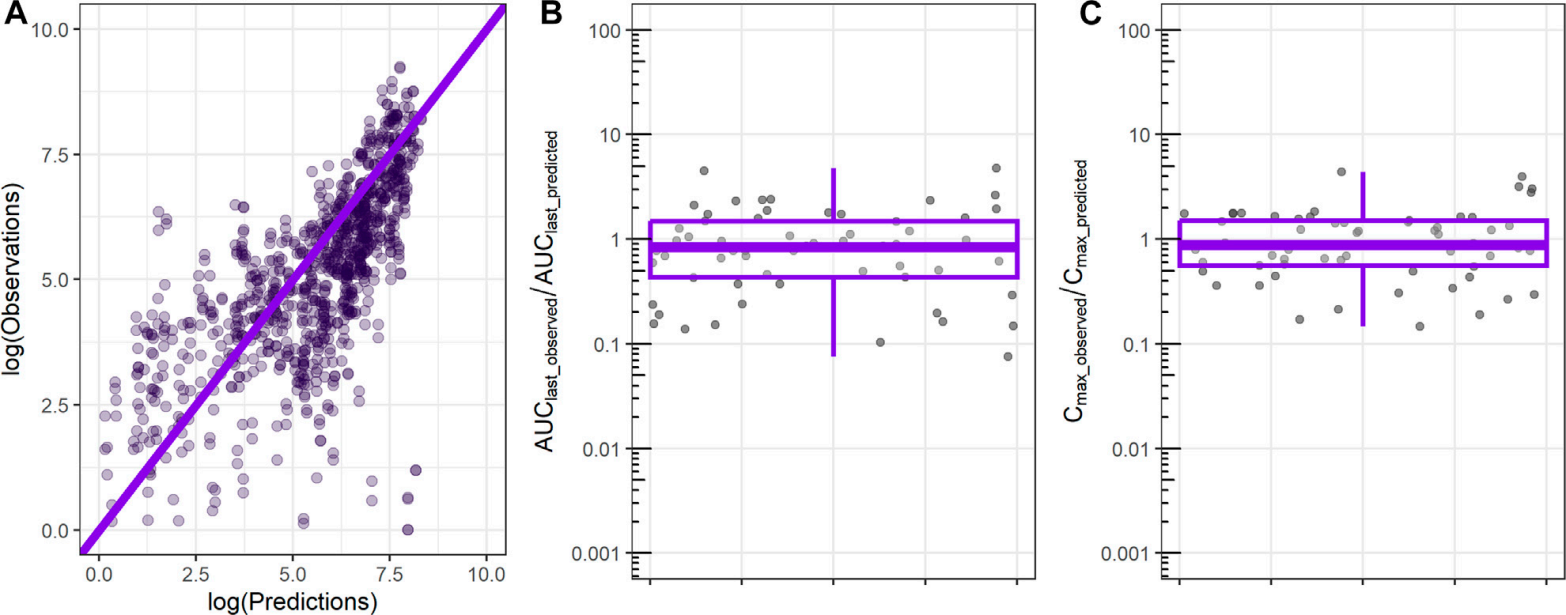
Comparison between observed rat plasma exposure and exposure predicted by the 1-compartment model using as input ML-derived CL, Vdss parameters. **(A)** Observed plasma exposure vs. 1-compartment model predictions. The solid line indicates the identity line. **(B)** Ratio between observed AUC until the final time point (AUC_last_observed_) and 1-compartment model-predicted AUC (AUC_last_predicted_). **(C)** Ratio between observed maximum concentration (C_max_observed_) and 1-compartment model-predicted maximum concentration (C_max_predicted_).

ML-derived *in vivo* CL and *in vivo* Vdss were used as input to the 1-compartment model, and the resulting profile was compared to the observed rat PK exposure data. Comparison between observed and predicted rat plasma exposure is shown in Figure 3. The majority of the predicted time points are close to the observed values as shown by the clustering of most of points around the identity line (Figure 3A). The ratio between the observed AUC until the last time point of 24 h (AUC_last_) and the predicted AUC_last_ has a median of 0.9, and 50% of values are between 0.4 and 1.5 (Figure 3B), which indicates satisfactorily prediction of the relevant metrics. Similarly, the ratio of the observed maximum concentration and the predicted C_max_ has an average of 0.9 and a 50% range narrower than the AUC_last_ ratio (Figure 3C).

**FIGURE 4:**
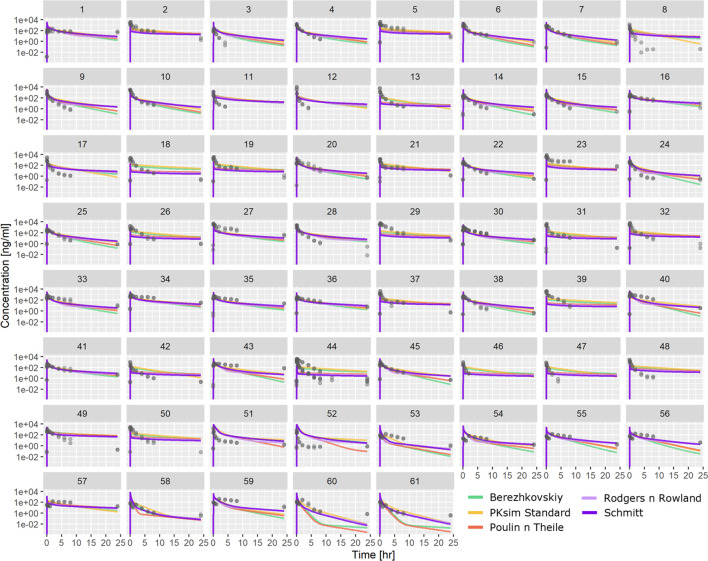
Comparison between observed PK profiles and profiles predicted using PBPK modeling with ML-driven PC/PK parameters and *in vivo* CL for the individual compounds tested. Different subplots indicate different compounds tested, and different colors indicate different distribution models.

PBPK-based predictions using ML-driven parameters (Table 1) were also tested against the same PK profile dataset. Figure 4 shows individual profiles along with PBPK-based predictions using *in vivo* CL for the different distribution models investigated. The PBPK model predictions appear to satisfactorily describe the majority of the compounds’ PK profile. The PBPK model was able to capture the bi-exponential nature of the compounds’ plasma distribution. Comparing the different distribution models, simulations show that there are cases where all of them conclude in similar exposure prediction (e.g., compound 5 and others), whereas, for cases such as compound 60 or 61 and others, the PK profile has significantly different characteristics depending on the distribution model used.

**FIGURE 5:**
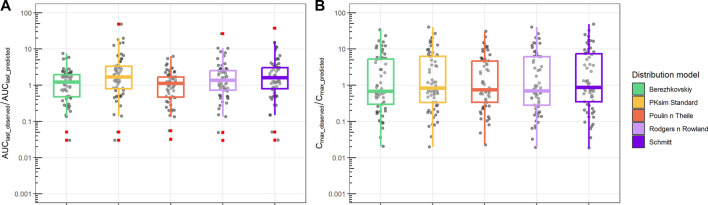
Ratio between observed and predicted AUC and C_max_ based on PBPK prediction using ML-driven PK/PC parameters and *in vivo* clearance (CL). **(A)** Ratio between observed AUC until the final time point (AUC_last_observed_) and PBPK model-predicted AUC (AUC_last_predicted_). **(B)** Ratio between observed maximum concentration (C_max_observed_) and PBPK model-predicted maximum concentration (C_max_predicted_). Different boxplot colors indicate different distribution models.

Analysis of the AUC_last_ and Cmax fold difference between observations and predictions for all PBPK distribution models is shown in Figure 5. Different distribution models have slightly different median values, but all distribution models tested showed a median AUC_last_observed_/AUC_last_predicted_ range between 1 and 2, which indicates a reasonable prediction of observed AUC. Similarly, the ratio between C_max_observed_ and C_max_predicted_ retained a median range of 0.5 to 0.8. Compared to the AUC ratio, Cmax retained a larger spread of the predicted values.

**FIGURE 6:**
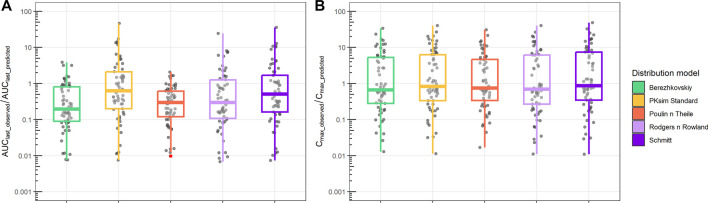
Ratio between observed and predicted AUC and Cmax based on PBPK prediction using ML-driven PK/PC parameters and intrinsic clearance (CL_int_). **(A)** Ratio between observed AUC until the final time point (AUC_last_observed_) and PBPK model-predicted AUC (AUC_last_predicted_). **(B)** Ratio between observed maximum concentration (C_max_observed_) and PBPK model-predicted maximum concentration (C_max_predicted_). Different boxplot colors indicate different distribution models.

Finally, we compared PBPK simulations using ML-driven PK/PC parameters and intrinsic clearance (CLint) with observed data (Figure 6). The median AUC_last_observed_/AUC_last_predicted_ ranged from 0.2 to 0.7. The interquartile difference was different between the distribution models used. In contrast, the C_max_observed_/C_max_predicted_ median had a narrower range (0.7–0.9) for all distribution models, with similar interquartile intervals.

## Discussion

In the past few decades, there have been considerable efforts to improve R&D productivity in the pharmaceutical industry. The main focus is to ensure that a molecule has desired pharmacological activity in humans prior to Phase II studies, with a heavy focus on having enough good Phase I-ready molecules (Paul et al., 2010). In this quick-win-fast-fail paradigm, ML and mechanism-based modeling have found increasing application to all stages of drug discovery and development (Pillai et al., 2022).

The application of ML is particularly appealing in the early stages of drug development where there is not enough mechanistic knowledge of how a new molecule distributes in the body and mediates its pharmacological effects. Several quantitative structure–activity relationship (QSAR) models for predicting preclinical ADME properties have been described (Van der Graaf et al., 1999; Ng et al., 2004; Gombar and Hall, 2013; Dave and Morris, 2015; Valitalo et al., 2016) and used in drug discovery. In this work, ML has been used to predict compounds’ PK/PC properties that are later used as input for mechanistic models to ultimately predict plasma exposure. Based on the ML models developed, it was found that, for all parameters except Fu, the average error in predictions with respect to observation was less than two-fold (MAPE values less than 1), while the average error for Fu was slightly higher than two-fold (MAPE values 1.1)—perhaps due to lack of data in the training set. We further assessed ML-driven propagation error in the ultimate exposure predictions by comparing 1-compartment/PBPK simulations using input parameters measured experimentally vs. predicted by ML (Supplementary Figure S2). Our analysis not only showed no significant difference in the PK profile prediction when using ML vs. experimental data, as indicated by overlapping boxplot edges, but it was also noted in some cases that ML models can mitigate errors that may occur due to variability in either recording or performing the experiment (Supplementary Figure S2, more outliers in data vs. ML predictions). The ML models developed in this study can be further improved by utilizing deep learning techniques and classical approaches when more data are available.

ML-derived *in vivo* CL and *in vivo* Vdss were initially used as input parameters to a 1-compartment model, and the resulting plasma exposure was tested against rat plasma profile (Figure 3A). The 1-compartment model is the simplest PK model that assumes a single volume of drug distribution (Vdss) with a linear clearance (CL); it is designed to explain mono-exponential PK profiles (straight line in logarithmic concentration vs. time plot). As such, 1-compartment representation is not suitable for capturing the distribution of the molecule to peripheral tissues of the body. Due to this inherent limitation, we observed a cluster of concentration data over the identity line at lower concentration values (Figure 3A), an indication of model underpredictability. Despite this inherent limitation, the 1-compartment model was able to capture the majority of both AUC_last,_ and C_max_ of observed data within a two-/three-fold difference (Figures 3B, C). In pharmacokinetics/toxicokinetics, there is no *a priori* threshold on an acceptable model error, but PBPK/PK models are generally accepted and considered useful when the prediction error on AUC or Cmax is in the two- or three-fold range (De Buck et al., 2007; Maharaj and Edginton, 2014; Mavroudis et al., 2018; Mavroudis et al., 2022).

To predict a new molecule’s tissue distribution in the absence of exposure data, quantitative pharmacologists often use PBPK modeling (Chen et al., 2012; Jones et al., 2015). The physiological details incorporated in PBPK models and the distribution models involved in their mathematical representation enable the prediction of tissue distribution based solely on the PC properties of the compound and the physiology of the relevant species. ML-driven parameters were used as input to the PBPK model (Table 1), considering all distribution models available in PK-Sim. In the results shown in Figures 4, 5, the PBPK model uses *in vivo* clearance (CL) resulting from NCA. As expected, the PBPK model can capture the distribution clearance and, as such, the bi-exponential exposure of the molecules (Figure 4) better than the 1-compartment model (Supplementary Figure S3). The median fold difference between observed and predicted AUC_last_ ranged 1–2 depending on the distribution model chosen. This is an indication of the different assumptions involved in the different distribution models that ultimately lead to different clearances and exposure predictions (Figure 4). In contrast, C_max_ is mainly determined by the compound’s PC properties and the body’s physiology, which was identical for all distribution models tested. As a result, the interquartile differences observed in Cmax are similar for the different distribution models and the narrower median value range (Figure 5B).

Our framework was also tested with PBPK model-based predictions using ML-driven parameters and intrinsic clearance (CLint) derived from *in vitro* experiments. For this case, although PBPK model could still satisfactorily describe the Cmax median within a two- to three-fold error (Figure 6B), the AUC_last_ prediction was significantly higher for the majority of the observed data, leading to a low AUC_observed_ over AUC_predicted_ ratio (Figure 6A; Supplementary Figure S4). This is a common observation and challenge across companies when using *in vitro* data (Petersson et al., 2022). In contrast with *in vivo* CL that results from NCA and represents a holistic representation of the *in vivo* PK data elimination rate, intrinsic clearance here solely represents the liver microsomal clearance. To be used in PBPK modeling, CL_int_ is scaled based on the relevant species liver weight, assuming a well-stirred liver compartment (Obach, 2001; Austin et al., 2002). In this case, all other clearance mechanisms (e.g., renal clearance) and active transports of the molecule (e.g., Pgp transporters) are not taken into consideration. This limited mechanistic representation of the molecule’s clearance pathways leads to an overprediction of AUC_last_ and, ultimately, lower values of AUC_last_ observed over a predicted ratio. Comparing AUC_last_ and Cmax predictions from all models and parameterizations used in this work using ML-driven input (Supplementary Figure S5), PBPK using CLint maintains a lower ratio of AUC_last_ observed/predicted, whereas PBPK with *in vivo* CL results in a similar AUC_last_ ratio prediction to the 1-compartment model. Cmax prediction shows significantly higher variation independent of *in vitro*/*in vivo* parameterization.

Due to recent progresses in bioinformatics and systems modeling, along with technical improvements in instrumentation and quantification methods that enable large numbers of molecules to be screened early in discovery, the methodology presented in this work has been investigated to some extent by others (Hosea and Jones, 2013) (https://www.simulations-plus.com/software/admetpredictor/). Compared to previous efforts, our analysis consists of a significantly larger test set of molecules and considers a number of alternative models and parameterizations; it is thus the first to examine the use of ML in combination with mechanistic modeling to such a conclusive extent. Henceforth, ML models may not be applied interchangeably in any given task due to their poor extrapolation capabilities outside the range of data utilized to train the model. Due to their black-box nature, ML models and methodologies need to be dynamically developed and fine-tuned in orchestration with new data generation, as is the case for the models presented in this work.

In conclusion, the ML/mechanistic modeling framework proposed here results in reasonable exposure predictions for most of the scenarios tested. Our work underlines the necessity of considering multiple distribution models when predicting PK based on molecular structure and providing the respective variability in addition to a single PK profile estimate. This effort aims to enable PK prediction earlier in the drug development process and ultimately help in prioritizing compounds for future evaluation.

## Data Availability

The data analyzed in this study are subject to the following licenses/restrictions: proprietary data that cannot be shared. Requests to access these datasets should be directed to panteleimon.mavroudis@sanofi.com.
