# Supplementary material for: Application of machine learning in combination with mechanistic modeling to predict plasma exposure of small molecules
Source: Front Syst Biol. 2023 Jun 20;3:1180948. doi: 10.3389/fsysb.2023.1180948 (PMC12342024; doi:10.3389/fsysb.2023.1180948)
Supplement: Supplementary file 1 [file DataSheet1.pdf]

Supplementary Material

Application of Machine Learning in combination with Mechanistic Modeling to Predict Plasma Exposure of Small Molecules

Panteleimon D. Mavroudis[1,*], Donato Teutonico[2], Alexandra Abos[3], Nikhil Pillai[1]

* **Correspondence:** Panteleimon D. Mavroudis: panteleimon.mavroudis@sanofi.com

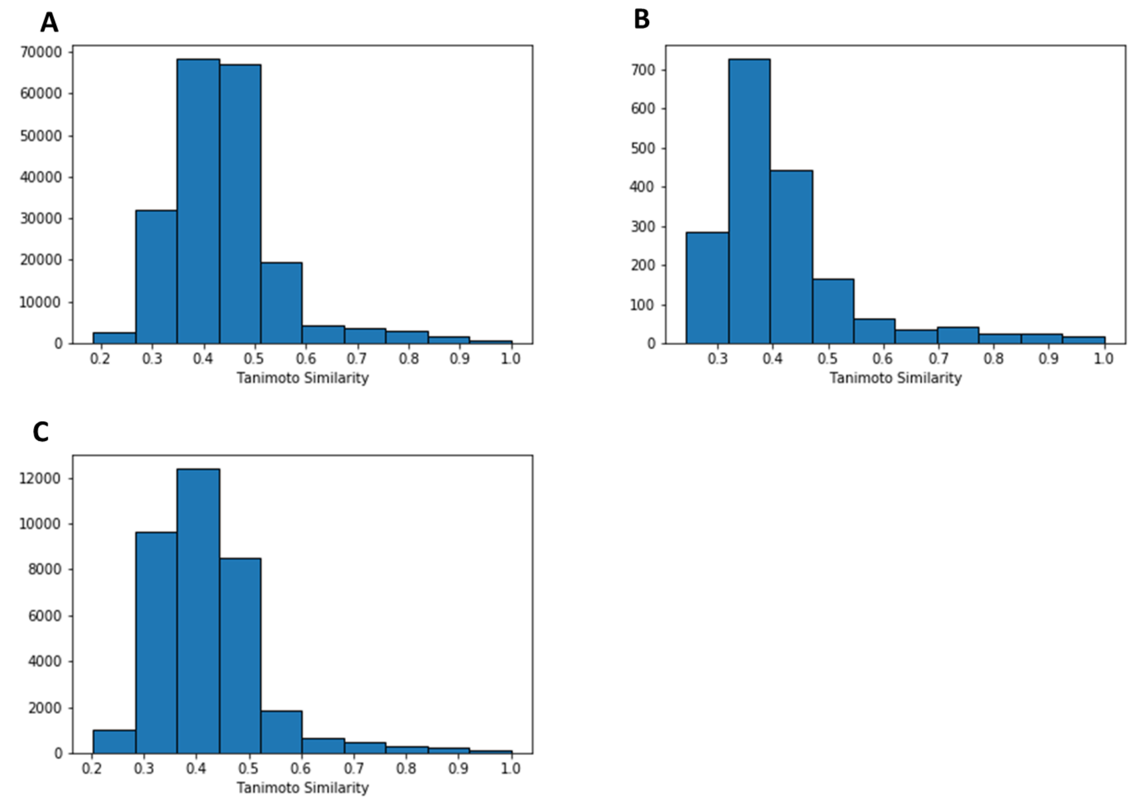

Supplementary Figure 1. Chemical similarity distribution based on Tanimoto score calculated based on RDKFingerprint generated using rdkit.Chem.Fingerprints.FingerprintMols module. A) For all compounds, N, B) For 61 compounds in the test set and C) For N-61 compounds vs 61 compounds. For the first two scenario's each compound was tested against every other compound in the same dataset, while in the final scenario compounds in the test set were compared against the compound which were not part of the test set. Most of the compounds in all 3 scenarios tested have Tanimoto score of 0.4 which suggest compounds' heterogeneity. Higher Tanimoto score(>0.8) suggest that the canonical forms for those compounds are similar, which may be possible when the compounds belong to the same family, which is quite common in initial stages of drug discovery where multiple compounds are generated to target the same target and disease.

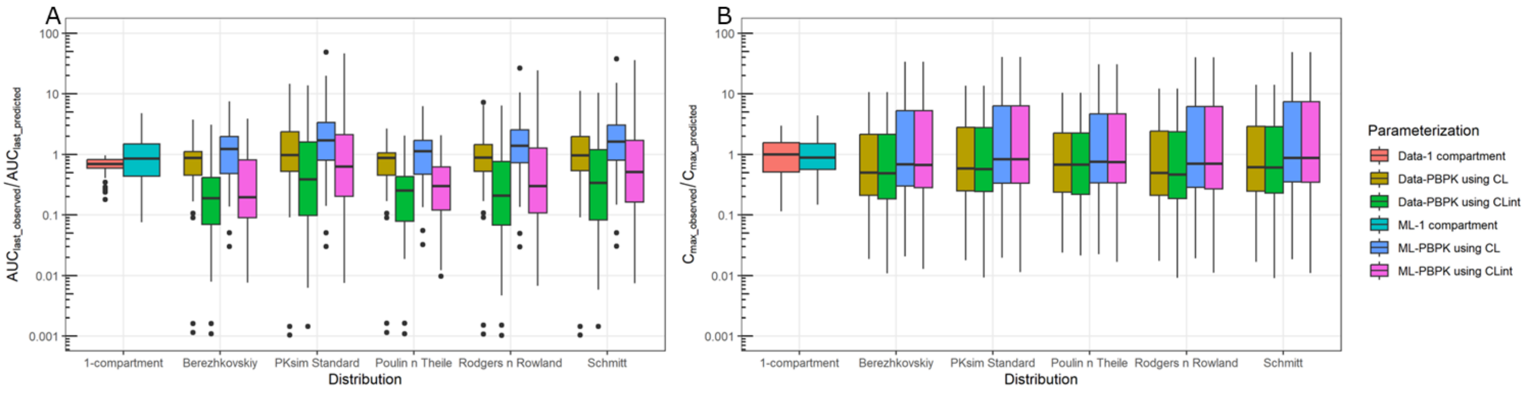

Supplementary Figure 2: Comparison between AUC_{last}, and C_{max} prediction resulting from 1-compartment and PBPK models using either experimentally measured (data) or ML driven properties, for different parameterizations (CL vs CLint). A) Ratio of AUC_{last} observed vs AUC_{last} predicted when different mechanistic models (1-compartment, PBPK) are informed using experimentally derived PK/PC data (Data) or ML predictions (ML). B) Ratio of C_{max} observed vs C_{max} predicted when different mechanistic models (1-compartment, PBPK) are informed using experimentally derived PK/PC data (Data) or ML predictions (ML). Data 1-compartment: Prediction using 1-compartment model informed from experimentally measured PK parameters. Data-PBPK using CL: Prediction using PBPK model with in-vivo CL informed from experimentally measured PC/PK parameters. Data-PBPK using CL_{int}: Prediction using PBPK model with intrinsic microsomal clearance (CLint) informed from experimentally measured PC/PK parameters. ML 1-compartment: Prediction using 1-compartment model informed from ML driven PK parameters. ML-PBPK using CL: Prediction using PBPK model with in-vivo CL informed from ML driven PC/PK parameters. ML-PBPK using CL_{int}: Prediction using PBPK model with intrinsic microsomal clearance (CLint) informed from ML driven PC/PK parameters,

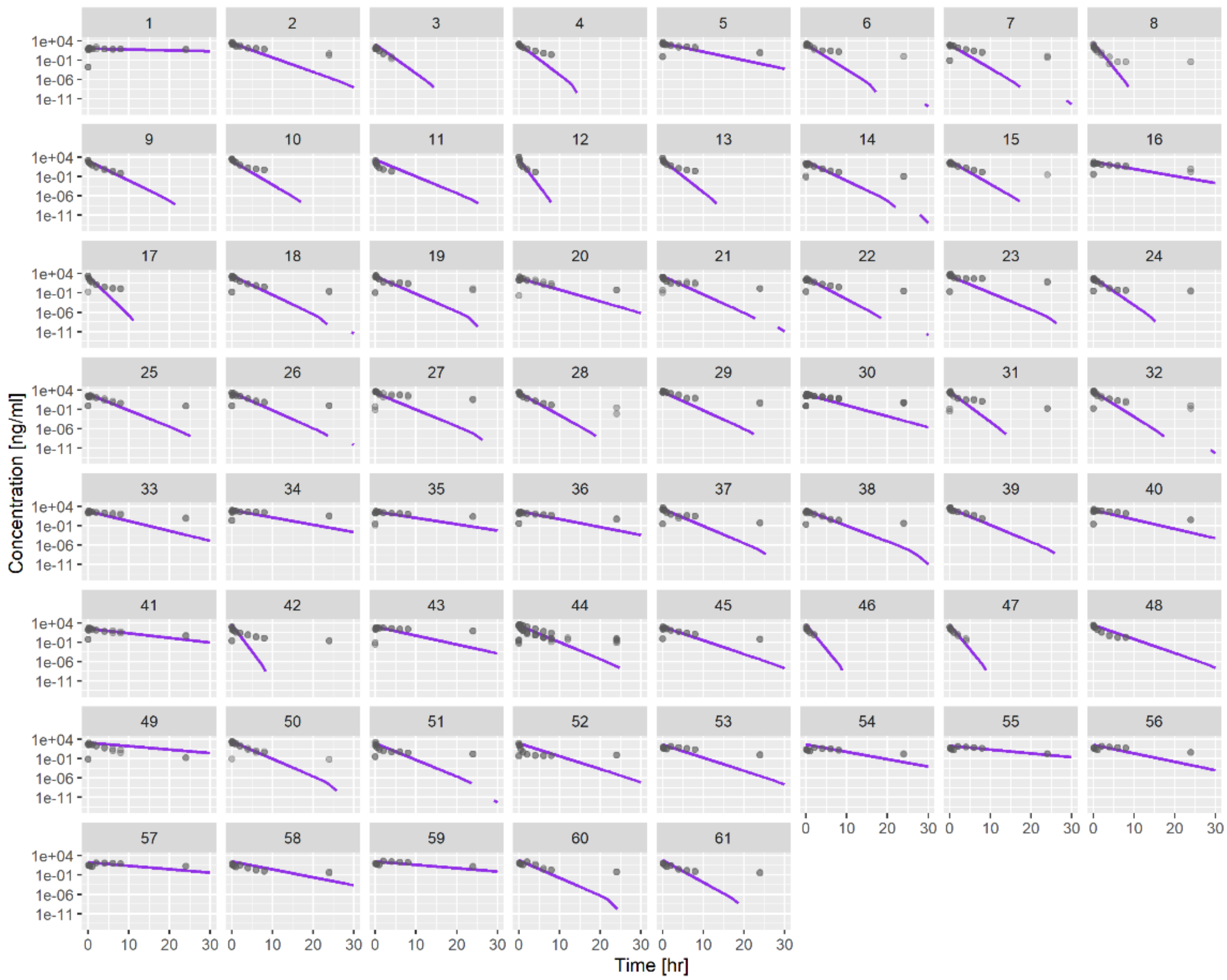

Supplementary Figure 3. Comparison between observed PK profiles and profiles predicted using 1-compartment modeling with ML driven PK parameters for the individual compounds tested. Different subplots indicate different compounds tested.

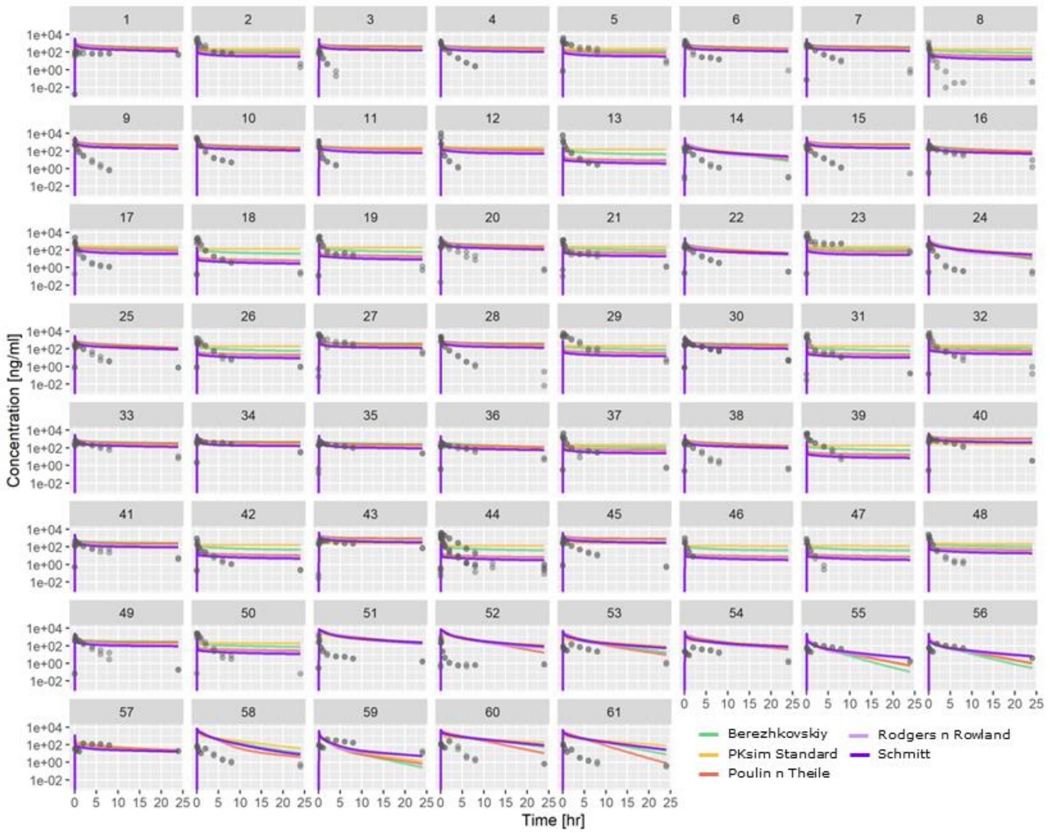

Supplementary Figure 4. Comparison between observed PK profiles and profiles predicted using PBPK modeling with ML driven PC/PK parameters and intrinsic CL_{int} for the individual compounds tested. Different subplots indicate different compounds tested and different colors indicate different distribution models.

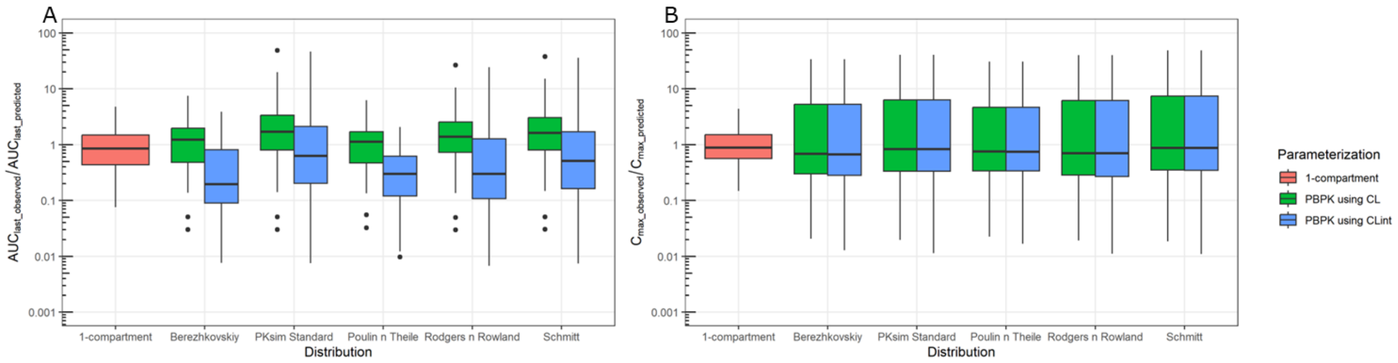

Supplementary Figure 5: Comparison between AUC_{last}, and C_{max} prediction resulting from 1-compartment and PBPK models using either in-vivo or in-vitro clearance parameterization (CL, CL_{int}) and ML driven input. A) Ratio of AUC observed vs AUC predicted for different mechanistic models tested with different parameterization (1-compartment, PBPK informed using in-vivo CL, PBPK informed using in-vitro CLint). B) Ratio of Cmax observed vs Cmax predicted for different mechanistic models. Models have been informed using machine learning predictions.

Parameter	Algorithm	MAPE	RMSE
CL	Random Forest on fingerprints	0.84	15.8
CL	XGBoost on fingerprints	0.82	15.74
CL	Support Vector Regression on fingerprints	0.99	16.56
CL	XGBoost on descriptors	0.81	17.28
CL	Support Vector Regression on descriptors	0.97	17
CL	Random Forest on descriptors	0.87	16.9
CL	Message passing neural network	0.7	18
Vdss	Random Forest on fingerprints	0.75	936
Vdss	XGBoost on fingerprints	0.72	946
Vdss	Support Vector Regression on fingerprints	0.74	1235
Vdss	XGBoost on descriptors	0.57	961
Vdss	Support Vector Regression on descriptors	0.76	1181.5
Vdss	Random Forest on descriptors	0.64	991
Vdss	Message passing neural network	0.7	2244
Clint	Random Forest on fingerprints	0.685	32.96
Clint	XGBoost on fingerprints	0.7	32.55
Clint	Support Vector Regression on fingerprints	0.83	31.75
Clint	XGBoost on descriptors	0.75	30.6
Clint	Support Vector Regression on descriptors	1.1	28.47
Clint	Random Forest on descriptors	0.89	32.7
CLint	Message passing neural network	0.5	50
pka basic	Random Forest on fingerprints	0.26	1.48
pka basic	XGBoost on fingerprints	0.32	1.5
pka basic	Support Vector Regression on fingerprints	0.28	1.47
pka basic	XGBoost on descriptors	0.3	1.41
pka basic	Support Vector Regression on descriptors	0.36	1.6
pka basic	Random Forest on descriptors	0.3	1.4

pka basic	Message passing neural network	0.3	3
pka acid	Random Forest on fingerprints	0.054	1.2
pka acid	XGBoost on fingerprints	0.077	1.24
pka acid	Support Vector Regression on fingerprints	0.12	1.58
pka acid	XGBoost on descriptors	0.07	1.7
pka acid	Support Vector Regression on descriptors	0.09	1.55
pka acid	Random Forest on descriptors	0.064	1.38
pka acid	Message passing neural network	0.1	2.5
fu	Random Forest on fingerprints	1.86	0.061
fu	XGBoost on fingerprints	1.4	0.064
fu	Support Vector Regression on fingerprints	1.33	0.058
fu	XGBoost on descriptors	1.27	0.053
fu	Support Vector Regression on descriptors	2.68	0.054
fu	Random Forest on descriptors	1.11	0.052
fu	Message passing neural network	1.5	0.1

Supplementary Table 1. MAPE and RMSE metrics for different algorithms tested on different input representation for different parameters.

Best model for each parameter by evaluating MAPE and RMSE of different models which were built based on combination of molecular representation and ML algorithm. Hyper-parameter corresponding to best model of CL which makes use of XGboost algorithm were n_estimators=1000, eta=0.005, subsample=0.7, colsample_bytree=0.8; Hyper-parameter corresponding to best model of V which makes use of XGboost algorithm were n_estimators=1100, eta=0.004, subsample=0.7, colsample_bytree=0.9; Hyper-parameter corresponding to best model of Clint which makes use of XGboost algorithm were n_estimators=900, eta=0.006, subsample=0.6, colsample_bytree=0.8; Hyper-parameter corresponding

to best model of Pka (most acid) which makes use of SVR algorithm were kernel="rbf", C=100, gamma="auto", epsilon=0.1; Hyper-parameter corresponding to best model of Pka (most basic) which makes use of Random Forest algorithm were n_estimators=100, max_depth=None; Hyper-parameter corresponding to best model of Fu which makes use of Random Forest algorithm were n_estimators=80, max_depth=None.

Parameter	Model 1	Model 2	Model 3	Model 4	Model 5	Model 6	Model 7	Model 8	Model 9	Model 10	Mean	SD
**Cl**	0.68	0.66	1.09	0.91	0.71	1.84	1.37	1.44	2.61	0.76	1.21	0.60
**pKa (most acid)**	0.68	0.66	1.09	0.91	0.71	1.84	1.37	1.44	2.61	0.76	1.21	0.60
**pKa (most basic)**	0.68	0.66	1.09	0.91	0.71	1.84	1.37	1.44	2.61	0.76	1.21	0.60
**Vdss**	0.66	0.80	0.70	0.58	0.75	0.95	0.53	0.66	0.84	0.47	0.69	0.14
**fu**	3.63	2.22	0.66	4.08	0.68	0.71	0.54	0.73	1.31	4.09	1.86	1.44
**clint**	3.63	2.22	0.66	4.08	0.68	0.71	0.54	0.73	1.31	4.09	1.86	1.44

Supplementary Table 2. MAPE from 10 fold cross validation for different models along with their mean and standard deviation.
